# A-Kinase Anchoring Protein 9 Promotes Gastric Cancer Progression as a Downstream Effector of Cadherin 1

**DOI:** 10.1155/2022/2830634

**Published:** 2022-10-22

**Authors:** Qiang Yan, Youliang Wu, Deguan Li, Yongxiang Li

**Affiliations:** Department of General Surgery, The First Affiliated Hospital of Anhui Medical University, No. 218 Jixi Road, Hefei 230022, Anhui, China

## Abstract

**Background:**

Genetic studies identified a dozen of frequently mutated genes in gastric cancer, such as cadherin 1 (CDH1) and A-kinase anchoring protein 9 (AKAP9). Of note, genetic alterations including depletion and amplification frameshift mutations of AKAP9 have been observed in 10–15% of gastric cancer patients. However, it is unknown of the expression and role of AKAP9 in gastric cancer. This study is aimed to characterize the expression and function of AKAP9 in gastric cancer.

**Methods:**

Using qRT-PCR, we analyzed the mRNA levels of AKAP9 in gastric cancer patient samples. We investigated the role of AKAP9 in gastric cancer by performing cell proliferation assay, transwell assay, and mouse xenograft assay.

**Results:**

AKAP9 was upregulated in gastric cancer patients. Overexpression of AKAP9 promoted cell proliferation, migration, and gastric tumor growth. Loss of CDH1 elevated AKAP9 mRNA and protein levels.

**Conclusion:**

Our study demonstrates that AKAP9 functions as an oncoprotein to promote gastric cancer cell proliferation, migration, and tumor growth. Moreover, we reveal a possible molecular link showing that AKAP9 is a critical effector downstream of CDH1 in gastric cancer.

## 1. Introduction

Gastric cancer, also known as stomach cancer, is still a serious health problem worldwide and is the fifth common cancer type with 5.6% incidence of total cancer. High incidence is particularly observed in East Asia. Although the incidence of gastric cancer has started to decline, it remains the third leading cause of cancer-related deaths worldwide, with 1,089,103 new cases and 768,793 death in 2020 [1]. Surgery with perioperative chemotherapy is the standard treatment for the primary gastric cancer [2]. However, over 50% of gastric cancer patients after surgery had relapse or develop distant metastases, with the overall survival less than 1 year [3]. Recently, survival benefits were achieved by the combination of trastuzumab with chemotherapy in HER2 positive gastric cancer patients, which account for 10%–25% of total gastric cancer [4]. Therefore, treatment of gastric cancers based on molecular genetic status is a promising strategy.

Like many other cancer types, gastric cancer is a heterogeneous disease [5]. According to the Lauren criteria, it can be divided into two main subtypes: diffuse gastric cancer (DGC) and intestinal gastric cancer (IGC) [6]. IGC is typically occurred in older people and is associated with environmental factors, such as H. pylori infection, smoking and alcohol, while DGC is prone to diagnose in young people and correlated with genetic alterations [7]. With extensive efforts in mapping the genetic landscape of gastric cancer in the past ten years, genetic alterations in a number of genes, such as cadherin 1 (CDH1), TP53, RHOA, CTNN1A, and CMTM2, have been highly associated with the development of DGC [8–10]. According to the TCGA project, gastric cancers were classified into four molecular subtypes: Epstein-Barr virus (EBV) positive, chromosomal instability (CIN), genomically stable (GS), and microsatellite instability (MSI) [11]. Similar to DGC, the GS is prevalent in young people.

Although 80–90% of gastric cancers are sporadic, approximately 1–3% of gastric cancers are inherited, named as hereditary diffuse gastric cancer (HDGC) with a high prevalence of DGC and lobular breast cancer [12]. Notably, up to 50% of sporadic DGC patients contain CDH1 somatic mutations [13] and approximately 25% of HDGC patients harbor CDH1 germline mutations that can be autosomal-dominantly inherited [14]. Apart from genetic alterations, loss of CHD1 expression through CDH1 promoter hypermethylation was also observed in more than 50% of DGC [15]. Of note, mutations and promoter hypermethylation lead to aberrant CDH1 function and are assumed to be pathogenic, including increased risk of gastric and lobular breast cancers, poorer prognosis, and survival rate [12, 16]. Therefore, CDH1 mutations/expression may represent a diagnostic or prognostic marker of gastric cancer.

CDH1 gene encodes E-cadherin protein that belongs to the cadherin family. As a transmembrane protein, E-cadherin is required for maintaining cell membrane ion channel activity and epithelial tissue integrity [17]. Reduced expression or loss of E-cadherin is frequently observed in many advanced cancers [18, 19]. Interestingly, in HDGC, germline mutations trigger CDH1 promoter hypermethylation in the wildtype allele, leading to downregulation of CHD1 [20]. Extensive studies demonstrate that E-cadherin functions as a tumor suppressor. Inactivation of CDH1 decreases cell-cell adhesion and activates a couple of oncogenic signaling pathway such as RhoA signaling, Wnt, and MAPK pathways, promoting cancer metastasis and recurrence [18, 21, 22].

Notably, by sequencing genomic DNA from 153 gastric cancer patients, a recent study identified 29 novel frequently mutated genes. Among them, A-kinase anchoring protein 9 (AKAP9) is the most mutated one with recurrent mutation in 14.9% of gastric cancers. Of note, both amplification and deletion were detected in AKAP9 genes [10]. Consistently, another study showed that somatic frameshift mutations in AKAP9 were occurred in 11.7% of gastric cancer and 17.7% colorectal cancers (CRC) with high microsatellite instability [23]. Moreover, AKAP9 is overexpressed in CRC patients and promotes CRC and tumor metastasis [24]. However, it is unknown what is the function of AKAP9 in gastric cancer. Here, we will explore the role of AKAP9 and its crosstalk with E-cadherin in gastric cancer progression.

## 2. Materials and Methods

### 2.1. Gastric Cancer Patient Samples

Patient samples were obtained from 31 gastric cancer patients undergoing surgical resection in 2019–2020. There are 18 males and 13 females with average age of 55.7 ± 1.02 years. Patients were fully informed and written consents were obtained before sample collection. The study was carried out under the protocol approved by the Institutional Research Ethics Committee at The First Affiliated Hospital of Anhui Medical University (356c5.d2).

### 2.2. Cell Culture

The gastric cancer cell lines NCI-N87 and SNU-1 were purchased from American Type Culture Collection (ATCC) and cultured with RPMI-1640 Medium (#10-040-CV, Corning, Corning, NY) supplemented with 10% fetal bovine serum (FBS, Gibco, Grand Island, NY). All cells grow in the incubator at 37°C and with 5% CO_2_. Lipofectamine 3000 was used to transfect cells following the manufacturer's instructions (#L3000008, Thermo Fisher, Waltham, MA). Lentivirus packaging was produced as previously described [25].

### 2.3. Plasmids and shRNA

Flag-AKAP9 used for ectopic expression of AKAP9 (OHu26045) and pcDNA3.1+/C-(K)-DYK used as empty vector (EV) were purchased from GenScript (Nanjing, China). Lentiviral shAKAP9 vectors (TRCN0000232465 and TRCN0000232463) were purchased from Sigma-Aldrich. Lentiviral shGFP vector was obtained from Addgene (#30323, Watertown, MA).

### 2.4. Western Blot

Cells were harvested by scraper and lysed with Triton buffer 150 mM NaCl, 1% Triton-X100, 0.1% SDS, 50 mM Tris pH8.0, and protease inhibitor cocktail (Thermo Fisher) at 4°C for 20 min. The supernatant was collected after centrifugation for 10 min. Supernatant was transferred to a new tube and the total protein concentration was measured using NanoDrop One spectrophotometer. Equal volume 2X Laemmli sample buffer (1610737, Bio-Rad, Hercules, CA) were added to the supernatant and then heated in 95°C for 10 min. Equal amount proteins were resolved by SDS-PAGE gel for western blot analysis. Antibodies against AKAP9 (1 : 1000), GAPDH (1 : 3000), E-cadherin (1 : 2000), and Rabbit secondary antibody (1 : 5000) were purchased from Abcam (Cambridge, MA). The western blot images were developed using chemiluminescence detection kit (WBKLS0500, Millipore, Billerica, MA) and the ChemiDoc Imaging System from Bio-Rad.

### 2.5. qRT-PCR

Total RNA was extracted using NucleoSpin RNA Plus XS kit, and 1 *μ*g total RNA was used for cDNA synthesis by PrimeScript RT-PCR kit (TaKaRa, Dalian, China). The mRNA levels were examined using SYBR Green Supermix kit (Bio-Rad). All these procedures were performed according to the manufacturer's instructions. The qPCR was performed using the CFX96 Touch Real-Time PCR Detection System under conditions: 30 seconds initial denaturation at 95°C, then 40 cycles of 10 seconds at 95°C, and 30 seconds at 60°C. AKAP-9 mRNA levels were normalized to GAPDH levels. The primers were adopted from previous study [24] and listed below.

AKAP9-forward: 5′-ACTCAAGGCACAGCATAAACAC-3′

AKAP9-reverse: 5′-GTTCTTCACTGCGTC CCAA-3′

GAPDH-forward: 5′-ACAGTCAGCCGCATCTTCTT-3′

GAPDH-reverse: 5′-GACAAGCTT CCCGTTCTCAG-3′

### 2.6. Cell Growth Curve

Cells (1 × 10^4^ per well) were seeded in 6-well plates and counted manually using hemocytometer under light microscope with 20× magnification every day. Three independent experiments were performed.

### 2.7. Transwell Migration Assay

Transwell assays were performed using inserts with 8.0 *µ*m pore membrane (Corning) in a 24-well plate. The cells (1 × 10^4^) were suspended in 100 *μ*l serum-free RPMI-1640 Medium and added to the upper chamber. The bottom of the well was refilled with 600 *μ*l RPMI-1640 Medium containing 10% FBS. After 24 h incubation, unmigrated cells on the top of the insert were scraped with a cotton swab. The migrated cells on the bottom of the membrane were fixed with 4% paraformaldehyde for 10 min and washed twice with phosphate buffered saline (PBS, pH 7.4). The migrated cells were incubated with 0.5% crystal violet solution for 15 min at room temperature and then were visual under microscope.

### 2.8. Mouse Tumor Xenograft Assay

The animal protocol was approved by The First Affiliated Hospital of Anhui Medical University. Five-week-old immunodeficient male mice were obtained from Vital River Laboratory Animal Technology (Beijing, China) and kept under conditions of 22°C and 12-h light/12-h dark cycle. A total of 2 × 10^6^ cells were injected subcutaneously into one side of mice. Tumor length (*L*) and width (*W*) were measured every four days. Tumor volumes were calculated by equation *L* × *W*^2^ × 0.52. At the day 31 (the endpoint), the mice were euthanized with CO_2_. Tumors were dissected and weighted. Tumors were fixed with 10% formalin for 24 h, then embedded in paraffin, processed, and mounted on the slides. Ki-67 staining was performed using anti-Ki-67 Rabbit antibody (Cell Signaling Technology, Danvers, MA).

### 2.9. Statistical Analysis

The RT-PCR assay, cell growth curve, and Transwell assay were independently performed three times. Significance was determined by ANOVA or Student's *t*-test. For xenograft mouse assays, five mice were used in each group. Data were presented as mean ± s.e.m. *P* < 0.05 was considered significant.

## 3. Results

### 3.1. The Expression of AKAP9 is Upregulated in Gastric Cancer

**Figure 1:**
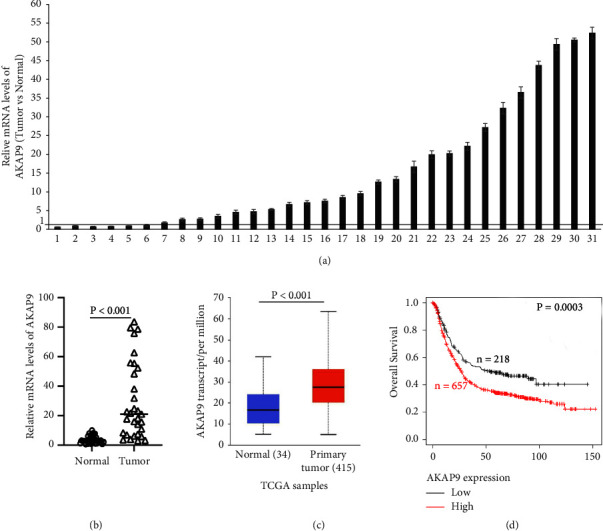
AKAP9 expression is elevated in gastric cancer samples. Analysis of AKAP9 mRNA levels by qRT-PCR in 31 gastric tumors. The AKAP9 mRNA levels were normalized to the paired adjacent normal tissues. (a-b) Examination of AKAP9 mRNA levels in 31 gastric tumors (Tumor) and matched adjacent normal tissues (Normal). (c) Comparison of AKAP9 transcription in primary tumors and normal tissues derived from TCGA database. (d) Analysis of the correlation between AKAP9 expression and overall survival in gastric cancer patients by Kaplan-Meier plotter.

Although AKAP9 is frequently mutated or amplified, its expression in gastric cancer remains unknown. We collected 31 gastric tumors and the matched adjacent normal tissues and examined AKAP9 mRNA levels. The qRT-PCR results showed that the mRNA levels of AKAP-9 in gastric tumors were higher than adjacent normal tissues in 25 of 31 matched tissue samples (Figures 1(a) and 1(b)). Consistently, the transcription of AKAP9 in TCGA samples was significantly increased in primary gastric tumors compared to normal tissues (Figure 1(c)), which was analyzed at http://ualcan.path.uab.edu/analysis.html [26]. To examine whether AKAP9 expression is associated with patient's prognosis, we analyzed the transcriptomic data of gastric cancers in NCBI GEO Database by Kaplan–Meier Plotter [27]. Notably, gastric cancer patients with high AKAP9 expression have shorter survival time than patients with low AKAP9 expression (Figure 1(d)). These results suggested that AKAP9 may play an important role in gastric cancer progression.

### 3.2. Knockdown of AKAP9 Inhibits Gastric Cancer Cell Proliferation and Migration

**Figure 2:**
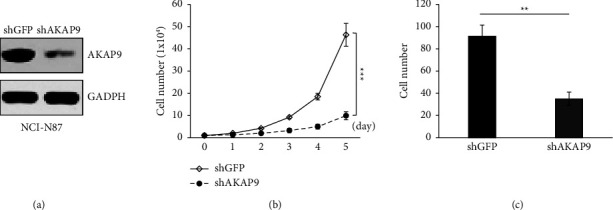
Downregulation of AKAP9 suppresses cell proliferation and migration. (a) Immunoblot examination of AKAP9 expression in NCI-N87 cells which were infected with shGFP or shAKAP9. (b) Examination of cell proliferation in NCI-N87 cells which were infected with shGFP or shAKAP9. ^*∗∗∗*^*P* < 0.001. (c) Quantitative analysis of migrated cells in Transwell assay using NCI-N87 cells which were infected with shGFP or shAKAP9. ^*∗∗*^*P* < 0.01.

To understand the role of AKAP9 in gastric cancer, we knocked down AKAP9 by infecting NCI-N87 gastric cancer cells with a mixture of two shRNAs targeting AKAP9 or shGFP lentivirus (as a negative control). Western blotting analysis confirmed a marked decrease of AKAP9 protein in shAKAP9 cells (Figure 2(a)). Importantly, cells depleted of AKAP9 displayed much slower proliferation than the control cells (Figure 2(b)). To test whether AKAP9 regulates cell migration, we performed Transwell assay and found that knockdown of AKAP9 significantly reduced cell migration capacity (Figure 2(c)). These results suggested AKAP9 is a positive regulator of gastric cancer cell proliferation and migration.

### 3.3. AKAP9 Overexpression Promotes Cell Proliferation and Migration

**Figure 3:**
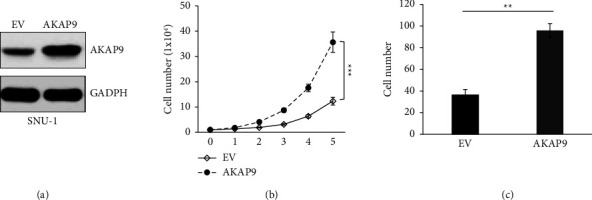
Overexpression of AKAP9 enhances cell proliferation and migration. (a) Immunoblot examination of AKAP9 expression in SNU-1 cells transfected with AKAP9 or EV. (b) Examination of cell proliferation in SNU-1 cells transfected with AKAP9 or EV. ^*∗∗∗*^*P* < 0.001. (c) Quantitative analysis of migrated cells in Transwell assay using SNU-1 cells transfected with AKAP9 or EV. ^*∗∗*^*P* < 0.01.

Having demonstrated that AKAP9 is upregulated in gastric cancer patients, we next examine if overexpression of AKAP9 has effects on gastric cancer cell proliferation and migration. We transfected SNU-1 cell with vector expressing AKAP9 or empty vector (EV) and validated upregulation of AKAP9 by western blotting (Figure 3(a)). In contrast to the results of AKAP9 knockdown, AKAP9 overexpression significantly enhanced cell proliferation and cell migration (Figures 3(b) and 3(c)), indicating that upregulation of AKAP9 may play a critical role in gastric cancer growth and metastasis.

### 3.4. AKAP9 Overexpression Promotes Tumor Growth in Mice

**Figure 4:**
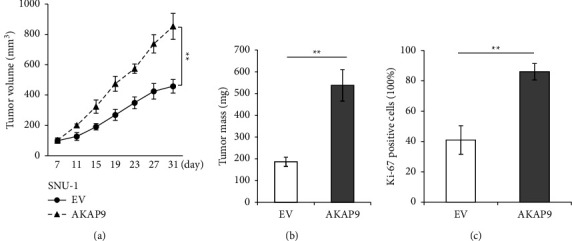
Overexpression of AKAP9 enhances tumorigenesis. (a) Mouse xenograft assays using SNU-1 cells expressing EV or AKAP9. Tumor size was measured every 4 days, and tumor volume was displayed. (*n* = 5 mice for each group). ^*∗∗*^*P* < 0.01. (b) Statistical analysis of tumor weight. ^*∗∗*^*P* < 0.01. (c) Statistical analysis of Ki-67 signal in tumors. Data were shown as mean ± s.e.m. ^*∗∗*^*P* < 0.01.

To investigate the effects of AKAP9 on tumor growth in vivo, we performed xenograft mouse assay by subcutaneously injecting the SNU-1 cells that express EV or AKAP9 into nude mice. We started to monitor tumor growth at 7-day post injection. During the tumor formation period, we found that tumor derived from AKAP9 expressing cells grew significantly faster than tumors derived from EV expressing cells (Figure 4(a)). At the end of this assay (31 days), the tumor size and weight were much higher in AKAP9 expressing group than EV expressing group (Figure 4(b)). Moreover, we examined cell proliferation by immunohistochemistry (IHC) staining of the Ki-67 in these tumors and found that tumors expressing AKAP9 displayed a significant higher Ki-67 signal than the tumors expressing EV (Figure 4(c)). These data demonstrated a critical role of AKAP9 in gastric tumorigenesis.

### 3.5. AKAP9 Is Required for CDH1 Loss-Mediated Gastric Cancer Cell Migration

**Figure 5:**
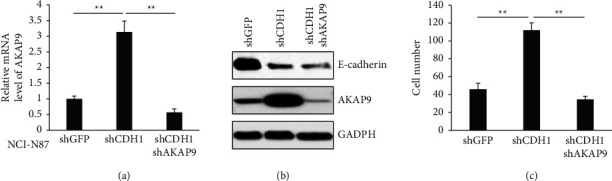
CDH1 regulates AKAP9 expression and cell migration. (a) Analysis of AKAP9 mRNA levels by qRT-PCR in NCI-N87 cells that were infected with shGFP or shCDH1 or shCDH1/shAKAP9. ^*∗∗*^*P* < 0.01. (b) Immunoblot analysis of AKAP9 protein levels in NCI-N87 cells that were infected with shGFP or shCDH1 or shCDH1/shAKAP9. (c) Quantitative analysis of migrated cells in Transwell assay using NCI-N87 cells that were infected with shGFP or shCDH1 or shCDH1/shAKAP9. ^*∗∗*^*P* < 0.01.

Given that both AKAP9 and CDH1 are frequently altered in gastric cancer and play a vital role in controlling gastric cancer cell migration, we next examine the correlation between these two functional-relevant events. Very interestingly, we found that knockdown of CDH1 by shRNA significantly increased both the mRNA and protein levels of AKAP9 in NCI-N87 cells, which could be suppressed by shAKAP9 (Figures 5(a) and 5(b)). Consistent with many previous studies summarized in the review [28], depletion of CDH1 significantly promoted cell migration in a Transwell assay, which could be reversed by AKAP9 silencing (Figure 5(c)). These data suggested that AKAP9 is a critical effector regulating CDH1-mediated gastric cancer migration or invasion.

## 4. Discussion

A-kinase anchoring protein (AKAP) family contains around 50 members in mammals, which function as scaffolding proteins [29]. They can assemble kinases and phosphatases into a single complex to modulate their substrate phosphorylation and physiological function. For example, AKAP79 interacted with both PKA and the calcium-dependent protein phosphatase PP2B to balance Ser845 phosphorylation of GluR1 [30]. Moreover, AKAPs play a role in several signaling pathways integration. A notable example is that AKAP-Lbc connects cAMP signaling with Rho, PKD, and MAPK pathways [31–33]. As a member of this family, AKAP9 also played a role in cAMP signaling. It anchors PKA and PDE4D3 to the centrosome, providing a unique platform for selective regulation of centrosomal cAMP/PKA signals [34]. AKAP9 also has been reported to mediate the crosstalk between cAMP and InsP3/Ca^2+^ signaling pathways in the brain. To better understand the physiological function of AKAP9, it will be important to further map the AKAP9 interacting proteins and signaling pathways.

Accumulating evidence indicates that AKAPs have many physiological roles and associate with various human diseases. A well-known physiological role of AKAPs is the regulation of cardiac functions including vascular integrity, peripheral arteries vasoconstriction, and hypertension. Dysregulation of AKAPs may lead to multiple cardiovascular diseases such as myocardial infarction, heart failure, and stroke [35]. Emerging studies have also demonstrated that AKAPs can function as oncogenic proteins or tumor suppressors to regulate cancer progression. Downregulation of gravin (AKAP12) was frequently observed in various cancers including prostate, ovarian, and breast cancers, and depletion of gravin promotes progression of these cancers [36]. Therefore, gravin is considered as a tumor suppressor. On the other hand, AKAP9 was reported to be upregulated in CRC and facilitate CRC progression by regulating Cdc42 interacting protein 4 expression [24]. In the current study, we found that AKAP9 is overexpressed in gastric cancer and enhances gastric tumorigenesis and metastasis. However, its downstream effectors have not been identified, which warranties a follow up study in the future.

Genomic studies demonstrated that genetic alteration in AKAPs is an important mechanism contributing to their aberrant functions and human diseases. The S1570L mutation in Yotiao (a splice variant of the AKAP9) disrupts its interaction with the cardiac potassium channel I_K_s and is associated with familial long-QT syndrome (LQTS) [37]. Moreover, two AKAP9 mutations (rs144662445 and rs149979685) were associated with Alzheimer disease by increasing Tau phosphorylation [38]. Notably, genetic alterations of AKAP9 are more complicated in gastric cancer, including depletion, amplification, and frameshift mutations [10, 23]. However, it is unknown whether these genetic alterations are associated with specific subtypes of gastric cancer. Moreover, the roles of these genetic alterations in gastric cancer have not been investigated. Answers to these questions represent important directions in future studies. Our study also provides an interesting finding that loss of CDH1 leads to elevation of AKAP9 expression. Given depletion of CDH1 enhances transcription factor *β*-catenin activity, it will be interesting to investigate that whether *β*-catenin or other transcription factors are involved in CDH1-mediated transcriptional regulation of AKAP9.

## 5. Conclusion

In conclusion, our study demonstrates that AKAP9 is overexpressed in gastric cancer and functions as an oncoprotein to promote gastric cancer cell proliferation, migration, and tumor growth. We also identify CDH1 as a potential negative regulator of AKAP9 expression, through which CDH1 regulates gastric cancer migration and invasion.

## Data Availability

Data could be obtained upon reasonable request to the corresponding author.

## References

[1] Sung H., Ferlay J., Siegel R. L., Laversanne M., Soerjomataram I., Jemal A. (2021). Global cancer statistics 2020: GLOBOCAN estimates of incidence and mortality worldwide for 36 cancers in 185 countries. *CA: A Cancer Journal for Clinicians*.

[2] Kong W.-J., Liu W.-D., Wang M., Hui W.-J., Gao F. (2022). Modulation of cisplatin resistance in gastric cancer by LncRNA SNHG15. *STEMedicine*.

[3] Orditura M., Galizia G., Sforza V., Gambardella V., Fabozzi A., Laterza M. M. (2014). Treatment of gastric cancer. *World Journal of Gastroenterology*.

[4] Bang Y. J., Van Cutsem E., Feyereislova A. (2010). Trastuzumab in combination with chemotherapy versus chemotherapy alone for treatment of HER2-positive advanced gastric or gastro-oesophageal junction cancer (ToGA): a phase 3, open-label, randomised controlled trial. *The Lancet*.

[5] Peng C., Kang W., Li Y. (2022). Respiratory chain complex I is related to oxidative phosphorylation in gastric cancer stem cells. *STEMedicine*.

[6] Lauren P. (1965). The two histological main types of gastric carcinoma: diffuse and so-calledintestinal-type carcinoma. An attempt at a histo-clinical classification. *Acta Pathologica et Microbiologica Scandinavica*.

[7] Cislo M., Filip A. A., Arnold Offerhaus G. J. (2018). Distinct molecular subtypes of gastric cancer: from Lauren to molecular pathology. *Oncotarget*.

[8] Kakiuchi M., Nishizawa T., Ueda H. (2014). Recurrent gain-of-function mutations of RHOA in diffuse-type gastric carcinoma. *Nature Genetics*.

[9] Cho J., Ahn S., Son D. S. (2019). Bridging genomics and phenomics of gastric carcinoma. *International Journal of Cancer*.

[10] Cai H., Jing C., Chang X. (2019). Mutational landscape of gastric cancer and clinical application of genomic profiling based on target next-generation sequencing. *Journal of Translational Medicine*.

[11] The Cancer Genome Atlas Research Network (2014). Comprehensive molecular characterization of gastric adenocarcinoma. *Nature*.

[12] Fitzgerald R. C., Hardwick R., Huntsman D. (2010). Hereditary diffuse gastric cancer: updated consensus guidelines for clinical management and directions for future research. *Journal of Medical Genetics*.

[13] Becker K. F., Atkinson M. J., Reich U. (1994). E-cadherin gene mutations provide clues to diffuse type gastric carcinomas. *Cancer Research*.

[14] Caldas C., Carneiro F., Lynch H. T. (1999). Familial gastric cancer: overview and guidelines for management. *Journal of Medical Genetics*.

[15] Machado J. C., Oliveira C., Carvalho R. (2001). E-cadherin gene (CDH1) promoter methylation as the second hit in sporadic diffuse gastric carcinoma. *Oncogene*.

[16] Corso G., Carvalho J., Marrelli D. (2013). Somatic mutations and deletions of the E-cadherin gene predict poor survival of patients with gastric cancer. *Journal of Clinical Oncology*.

[17] van Roy F., Berx G. (2008). The cell-cell adhesion molecule E-cadherin. *Cellular and Molecular Life Sciences*.

[18] Bracke M. E., Van Roy F. M., Mareel M. M. (1996). The E-cadherin/catenin complex in invasion and metastasis. *Current Topics in Microbiology and Immunology*.

[19] Carneiro F., Huntsman D. G., Smyrk T. C. (2004). Model of the early development of diffuse gastric cancer in E-cadherin mutation carriers and its implications for patient screening. *The Journal of Pathology*.

[20] Barber M., Murrell A., Ito Y. (2008). Mechanisms and sequelae of E-cadherin silencing in hereditary diffuse gastric cancer. *The Journal of Pathology*.

[21] Klezovitch O., Vasioukhin V. (2015). *Cadherin signaling: keeping cells in touch F1000 Faculty Rev*.

[22] Yu W., Yang L., Li T., Zhang Y. (2019). Cadherin signaling in cancer: its functions and role as a therapeutic target. *Frontiers in Oncology*.

[23] Jo Y. S., Kim M. S., Yoo N. J., Lee S. H. (2016). Frameshift mutations of AKAP9 gene in gastric and colorectal cancers with high microsatellite instability. *Pathology and Oncology Research*.

[24] Hu Z. Y., Liu Y. P., Xie L. Y. (2016). AKAP-9 promotes colorectal cancer development by regulating Cdc42 interacting protein 4. *Biochimica et Biophysica Acta - Molecular Basis of Disease*.

[25] Tiscornia G., Singer O., Verma I. M. (2006). Production and purification of lentiviral vectors. *Nature Protocols*.

[26] Chandrashekar D. S., Bashel B., Balasubramanya S. A. H. (2017). UALCAN: a portal for facilitating tumor subgroup gene expression and survival analyses. *Neoplasia*.

[27] Szasz A. M., Lanczky A., Nagy A. (2016). Cross-validation of survival associated biomarkers in gastric cancer using transcriptomic data of 1, 065 patients. *Oncotarget*.

[28] Liu X., Chu K. M. (2014). E-cadherin and gastric cancer: cause, consequence, and applications. *BioMed Research International*.

[29] Langeberg L. K., Scott J. D. (2005). A-kinase-anchoring proteins. *Journal of Cell Science*.

[30] Tavalin S. J., Colledge M., Hell J. W., Langeberg L. K., Huganir R. L., Scott J. D. (2002). Regulation of GluR1 by the A-kinase anchoring protein 79 (AKAP79) signaling complex shares properties with long-term depression. *Journal of Neuroscience*.

[31] Carnegie G. K., Smith F. D., McConnachie G., Langeberg L. K., Scott J. D. (2004). AKAP-Lbc nucleates a protein kinase D activation scaffold. *Molecular Cell*.

[32] Smith F. D., Langeberg L. K., Cellurale C. (2010). AKAP-Lbc enhances cyclic AMP control of the ERK1/2 cascade. *Nature Cell Biology*.

[33] Diviani D., Soderling J., Scott J. D. (2001). AKAP-lbc anchors protein kinase A and nucleates g*α*12-selectiverho-mediated stress fiber formation. *Journal of Biological Chemistry*.

[34] Terrin A., Monterisi S., Stangherlin A. (2012). PKA and PDE4D3 anchoring to AKAP9 provides distinct regulation of cAMP signals at the centrosome. *Journal of Cell Biology*.

[35] Diviani D., Reggi E., Arambasic M., Caso S., Maric D. (2016). Emerging roles of A-kinase anchoring proteins in cardiovascular pathophysiology. *Biochimica et Biophysica Acta (BBA) - Molecular Cell Research*.

[36] Gelman I. H. (2012). Suppression of tumor and metastasis progression through the scaffolding functions of SSeCKS/Gravin/AKAP12. *Cancer & Metastasis Reviews*.

[37] Chen L., Marquardt M. L., Tester D. J., Sampson K. J., Ackerman M. J., Kass R. S. (2007). Mutation of an A-kinase-anchoring protein causes long-QT syndrome. *Proceedings of the National Academy of Sciences of the United States of America*.

[38] Ikezu T., Chen C., DeLeo A. M. (2018). Tau phosphorylation is impacted by rare AKAP9 mutations associated with alzheimer disease in african Americans. *Journal of Neuroimmune Pharmacology*.

